# Adult Post-operative Mental Health Outcomes Following Abdominal and Orthopedic Surgeries: A Scoping Review

**DOI:** 10.7759/cureus.115806

**Published:** 2026-09-05

**Authors:** Gilbert Mua Akwa, Nale Christine Tiemah, Achangwa Chabeja, Franklin Chu Buh, Marios Kantaris

**Affiliations:** 1 Department of Public Health, Faculty of Medicine and Biomedical Sciences, University of Yaoundé I, Yaounde, CMR; 2 Department of Public Health, Institute of Public Health, Liverpool John Moores University, Liverpool, GBR; 3 School of Biosciences, University of Kent, Canterbury, GBR; 4 Department of Physiotherapy and Physical Medicine, University of Dschang, Douala, CMR; 5 Department of Public Health, Health Services and Social Policy Research Centre, Larnaca, CYP

**Keywords:** abdominal surgery, orthopedic surgery, post-operative anxiety, post-operative depression, post-traumatic stress disorder

## Abstract

Poor mental health has been shown to negatively impact surgical outcomes; however, the impact of surgery on mental health remains under-investigated. Therefore, in this scoping review, we aimed to map the breadth and depth of evidence regarding post-operative mental health challenges faced by surgical patients. The review was conducted and reported in accordance with the Joanna Briggs Institute (JBI) methodology and the Preferred Reporting Items for Systematic reviews and Meta-Analyses extension for Scoping Reviews (PRISMA-ScR) checklist. A comprehensive search was conducted across PubMed, PsycINFO, Web of Science, the Cochrane Library, and gray literature sources. Of 29,536 studies initially identified, 86 primary research studies on adult populations where the primary outcomes were post-operative depression, anxiety, or post-traumatic stress disorder (PTSD) were included. Most studies were conducted in China (21) and the USA (14). Depression and anxiety symptoms were highly prevalent among patients; levels typically peaked during the early post-operative period before gradually improving, with some studies reporting prevalence rates of >60%. However, marked improvements in anxiety and depressive symptoms were observed following bariatric and corrective orthopedic surgeries. Notably, PTSD was more common among amputees and women undergoing cesarean deliveries. Our findings highlight a scarcity of evidence from low-income countries, as well as a lack of data on PTSD in patients undergoing urological or abdominal procedures other than cesarean sections. Additionally, marked heterogeneity in mental health assessment tools complicates the synthesis, interpretation, and comparison of data across studies. Large-scale, multinational studies are rare. In conclusion, post-operative depression, anxiety, and PTSD commonly occur after abdominal and orthopedic surgeries, and can significantly impair the quality of recovery and rehabilitation. Consequently, a multidisciplinary, patient-centered approach is essential to optimize surgical and mental health outcomes, ultimately ensuring the delivery of holistic patient care. Also, there is an urgent need to develop and harmonize mental health assessment tools for the post-surgical setting.

## Introduction and background

Mental health is an essential component of psychological well-being that enables individuals to cope with adversity, function effectively, and participate actively in their communities. Globally, more than 1 billion individuals live with a mental health condition [1]. Severe life stressors, ranging from acute medical events such as myocardial infarction to major interventions such as coronary artery bypass surgery, can lead to severe psychiatric conditions, including major depression, anxiety, and post-traumatic stress disorder (PTSD) [2]. Consequently, surgery must be recognized not only as a physiological disruption but also as a significant psychological stressor [3]. According to Hsu et al., surgical patients are highly susceptible to pre-operative anxiety, post-operative depression, and PTSD [4]. Therefore, integrating mental health services into routine surgical care is essential to facilitate holistic recovery. Conversely, surgical intervention may also yield psychiatric benefits; a cohort study evaluating patients with refractory epilepsy demonstrated that those who underwent successful surgical management experienced a significant reduction in psychiatric symptoms one year after intervention compared with counterparts deemed unsuitable for surgery [5]. Nevertheless, adverse psychological outcomes remain pervasive, with 64% of hospitalized post-surgical patients experiencing depression, and 74% contending with severe anxiety. Furthermore, 13%-47% of patients navigate post-operative depression, a burden frequently exacerbated by physical discomfort, financial constraints, or a deficit in psychosocial support [6].

A systematic review and meta-analysis of 50 studies focusing on patients who developed post-surgical complications revealed that two-thirds of the patients experienced adverse psychological outcomes, even after controlling for pre-surgical psychological status. Furthermore, a one-year follow-up demonstrated that participants across half of the included studies exhibited poor psychological well-being [7]. Complementing these findings, a prospective study focusing on post-surgical patients with pre-existing severe mental illness revealed that a post-operative mental health visit significantly reduced the odds of hospital readmission. These findings highlight surgery as a potential psychological stressor, while highlighting the critical importance of post-operative mental healthcare in optimizing overall surgical outcomes [8]. Numerous studies have illustrated the detrimental impact of trauma and orthopedic interventions on psychological outcomes, specifically depression [9,10], anxiety [11,12], and PTSD [13,14]. This vulnerability is not restricted to adults; a cohort study evaluating pediatric patients who underwent adenoidectomy or tonsillectomy revealed a significant increase in stress-related conditions, such as PTSD, relative to their matched, unexposed counterparts [15].

Many research works have highlighted the impact of post-operative mental health on surgical outcomes, and some have delved further to uncover the post-operative challenges and poor outcomes recorded among patients with a history of pre-surgical severe mental illness [16-19]. The relationship between surgery and mental health has not been fully established [4]. Therefore, we aimed to map the breadth and depth of evidence on the effects of surgery on mental health challenges. Specifically, we aimed to elaborate on the tools used to evaluate mental health, identify gaps in the literature, elaborate on various emerging diagnostic and therapeutic approaches, and explore the factors influencing these mental health conditions.

## Review

Methods

Study Design

This study was conducted and reported in accordance with the Joanna Briggs Institute (JBI) manual and the Preferred Reporting Items for Systematic Reviews and Meta-Analyses extension for Scoping Reviews (PRISMA-ScR) reporting checklist, respectively. This scoping process was designed to comprehensively map the breadth and depth of the existing evidence in this field, establish a rigorous foundation for future primary studies, identify critical knowledge gaps, and clarify the core concepts and definitions used in the literature. Methodological quality and risk-of-bias assessments were not conducted because critical appraisal is not a mandatory component of scoping reviews [20]. The execution of this study followed a structured, sequential workflow: defining and refining the research query, establishing strict inclusion and exclusion criteria, performing a comprehensive literature search and article selection, extracting and analyzing data, and synthesizing and presenting the final insights.

Elaboration of Review Framework

The review question was structured using the Population (P: adult), Concept (C: mental health outcomes), Context (C: abdominal or orthopedic surgery) framework recommended by the JBI.

Eligibility Criteria

Inclusion criteria: We included original research articles that (1) evaluated adult patients aged ≥18 years who underwent a major abdominal or orthopedic surgery; (2) investigated the development of a post-operative mental health condition, specifically depression, anxiety, or PTSD as primary outcomes; (3) assessed these psychological outcomes perioperatively or up to two years following surgery; (4) were published in the English language; and (5) were indexed in the databases between January 2000 and May 2026.

Exclusion criteria: We excluded (1) studies exclusively evaluating well-established or pre-existing mental health conditions before surgery, (2) publications incorporating pediatric mental health outcomes following surgical interventions, (3) studies for which the complete abstract, full-text article, or post-operative monitoring timeline could not be retrieved, (4) research proposals or protocols outlining intended mental health outcomes; (5) and articles on mental health outcomes in patients undergoing spine and abdominal vascular surgeries.

Information Sources and Search Strategy

A comprehensive search approach was adopted, and information was retrieved from PubMed, PsycINFO, the Cochrane Library, and Web of Science, alongside gray literature sources such as the CDC, WHO, and relevant NGO publications. Search themes were combined using Boolean operators (AND/OR/NOT), using "AND" to intersect distinct themes, "OR" to capture alternative terminologies within a theme, and "NOT" to exclude irrelevant themes. We manually screened the reference lists of all included publications and related systematic review titles to identify additional articles relevant to our topic, thereby enhancing search sensitivity and specificity. The search strategy was continuously refined to optimize data retrieval. The literature search was performed from April to May 2026 by two independent reviewers. Conflicts were resolved by a third reviewer, and particularly difficult decisions were discussed with the entire review team until a consensus was reached.

Keywords and controlled vocabulary were systematically derived for each domain. To maximize search sensitivity, Medical Subject Headings (MeSH) terms were paired with text words, truncations, and wildcard characters. Individual search components were developed into four discrete keyword groups (#1, #2, #3, and #4) within the advanced search builder. These independent groups were subsequently merged in the search history using the Boolean operator AND to execute the final comprehensive search (#1 AND #2 AND #3 AND #4).

#1: "Adult/psychology"[Mesh] OR adult* OR elder* OR middle age; #2: abdominal surg* OR abdominal procedur* OR cholecystectom* OR pancreatectom* OR appendectom* OR gastrectom* OR laparotom* OR perforation repair OR intestinal anastomosis OR bariatric surg* OR by-pass surgery OR laparoscop* OR colectom*, hepatectom* OR hysterectom* OR gastrointestinal cancer resection OR joint replacement OR arthroplasty OR fracture repair OR fracture fixation OR osteosynthesis OR amputation OR cesarean delivery OR cesarean section OR myomectom* OR ovarectom* OR hysterectom* OR nephrectom* OR cystectom* OR ureteroplast* OR urological procedures OR urolological surg*; #3: Mental health OR psychological well-being OR emotional well-being OR mood disorders OR depression OR anxiety OR post-traumatic stress disorder OR psychosocial OR psychiatric; #4: outcome OR following surgery OR after surgery OR postoperative, perioperative.

Selection of Studies

**Figure 1:**
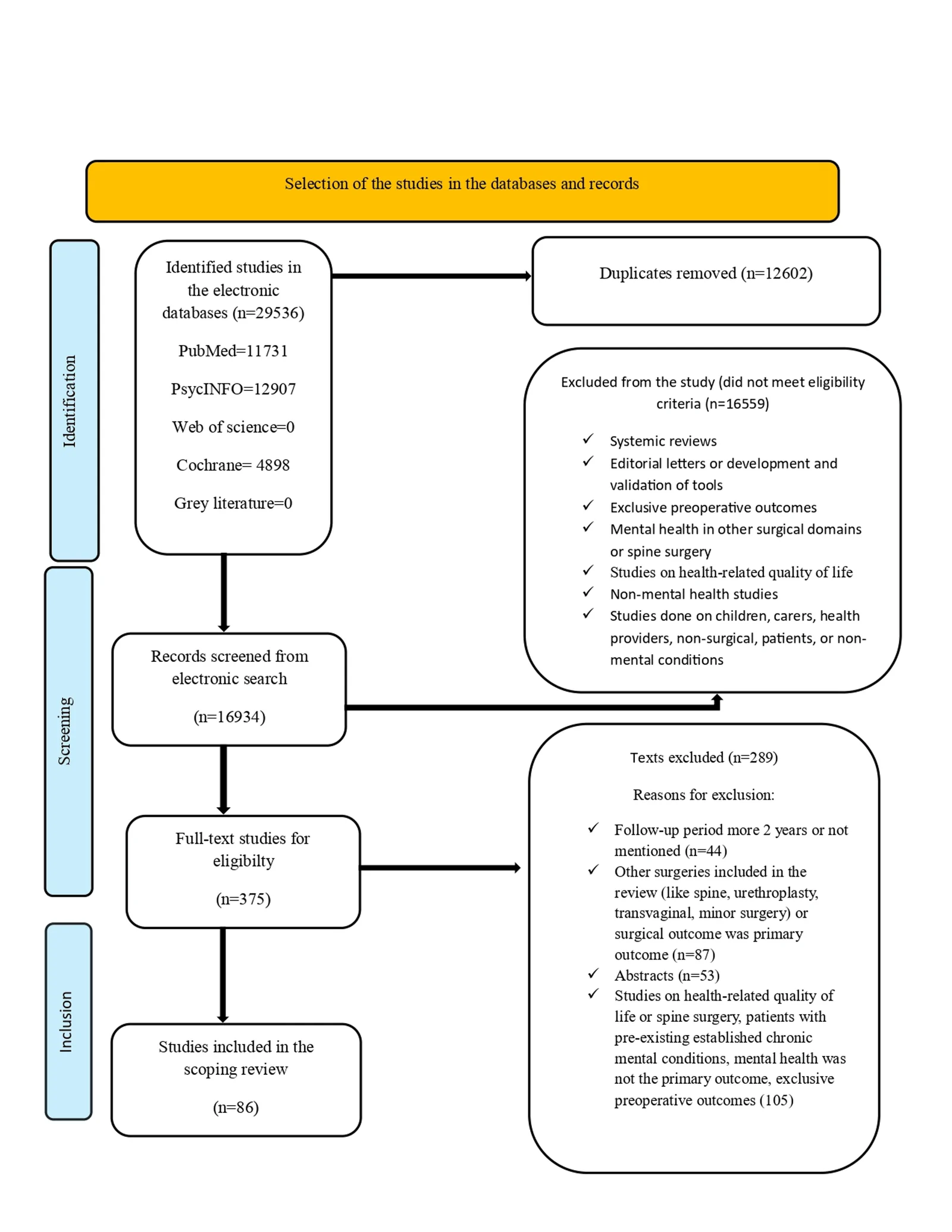
PRISMA-ScR flow diagram PRISMA-ScR, Preferred Reporting Items for Systematic reviews and Meta-Analyses extension for Scoping Reviews.

Citations of interest were imported into Zotero for deduplication. Subsequently, we carried out a two-stage screening process, consisting of a title and abstract screening followed by a full-text review. Initially, titles and abstracts were screened against the pre-established eligibility criteria. Subsequently, a comprehensive full-text review was performed on all potentially eligible articles to confirm their suitability for final inclusion. Any discrepancies or doubts regarding study eligibility were resolved by consulting a third reviewer. This study selection process was visually mapped using a PRISMA-ScR flow diagram (Figure 1).

A piloted data charting form was used to extract relevant information from the included studies, including author name(s), year of publication, country where the research was conducted, study aim, sample size, clinical characteristics of the study population, methodology, data analysis techniques, and primary outcomes.

Analysis and Synthesis of Data

Descriptive and thematic approaches were used to analyze the extracted data. The distribution of the included studies was subsequently mapped numerically to illustrate trends based on geographical location, publication year, study design, and target population group.

Quality Appraisal

A quality assessment of the individual included studies was not conducted, as it is mandatory for a scoping review. However, we described the inherent limitations of the included studies to facilitate a more nuanced interpretation of the findings.

Ethical Clearance

Formal institutional ethical approval was not required, as this study was a scoping review of secondary data from previously published literature rather than a primary investigation involving human participants. Nevertheless, strict adherence to research integrity and publication ethics was maintained by ensuring that all primary sources were meticulously cited and accurately referenced.

Results

Study Selection

The initial database search yielded 29536 records from PubMed (11731), PsycINFO (12907), Web of science (0), Cochrane (4898) and gray literature (0). After excluding 12602 duplicates, 16934 abstracts and titles were assessed for eligibility and 375 articles were retained for full-text review.

Most studies excluded during the full-text review were abstracts for which we could not find the full-text, studies centered on a broader domain like health-related quality of life (HRQOL) after surgery or on surgical outcome and not mental outcome, studies whose follow-up period exceeded two years, or studies, including surgical procedures, that were not in our scope despite sharing same specialty, such as breast surgery, urethroplasty, vagina procedures. We also excluded studies involving spine surgeries, even though they are sometimes performed by orthopedic surgeons. After the full-text review, 86 studies that met the inclusion criteria were included in the final review. Table 1 shows the data extraction chart of the included studies.

**Table 1:** Data extraction form of the included studies

Authors	Year	Country	Aim/purpose of the study	Sample size and population	Methodology	Findings
Economos et al. [21]	2023	France	To evaluate the epidemiology of depressive mood and anxiety for the six months following cytoreductive surgery (CRS) with or without hyperthermic intraperitoneal chemotherapy (HIPEC)	115 patients undergoing CRS with or without HIPEC	Prospective cohort. The same patients before and after surgery constituted the 2 arms. They were assessed before surgery and at every follow-up visit using a Hospital Anxiety and Depression Scale (HADS). A score >7 was diagnostic.	The incidence of a score > 7 was 0.05 per person-month. Overall, 32% had a score above 7. There was a significant increase in the average score, from 4.36 before surgery to 7.8 after surgery.
Morgan et al. [22]	2020	Australia	To analyze the relationship between bariatric surgery and the incidence of emergency department (ED), outpatient, and inpatient mental health service use	24,766 patients who underwent index bariatric surgery	Population-based, retrospective, longitudinal, mirror-image cohort study. With bariatric surgery as the exposure, the same patients, before and after surgery, constituted the two cohorts. The use of mental health services before and after the surgery was the primary outcome.	One in every six patients used a mental health service in the peri-operative period. There was a significant association between surgery and increased use of mental health services for conditions such as mood disorders, substance use disorders, and schizophrenia.
Alshammari et al. [23]	2022	Saudi Arabian	To assess the effect of bariatric surgery on the onset and exacerbation of anxiety and depression	367 bariatric surgery patients	A cross-sectional study was conducted involving patients who underwent bariatric surgery in the past two years. They were contacted by phone and asked to complete the Patient Health Questionnaire-9 (PHQ-9) and Generalized Anxiety Disorder-7 (GAD-7) for depression and anxiety, respectively.	The degree of anxiety, from mild to severe, was inversely proportional to the percentage of people affected (20.7%, 11.2%, and 8.7%). Similarly for depression: extremely mild (46.9%), mild (29.4%), moderate (11.2%), moderately high (8.2%), and severe depression (4.4%). There was no difference before and after surgery.
Osinowo et al. [24]	2003	Nigeria	To investigate the level of anxiety and depression before and after surgery and the effect of cognitive therapy among surgical patients in Nigeria	33 elective surgery patients (8 gynecologic and 25 general surgery)	Randomized controlled clinical trial. Patients were randomized into self-instructional training (SIT), control, and rational emotive therapy (RET) groups. They were administered the HADS and the State-Trait Anxiety Inventory (STAI) preoperatively and 24 hours postoperatively, after receiving either no therapy, SIT, or RET.	RET reduced post-operative depression, and SIT reduced post-operative anxiety.
Dong et al. [25]	2019	China	To evaluate in colorectal cancer patients undergoing post-operative chemotherapy complicated by depression, the efficacy of telephone-based RT (TBR) on depression, anxiety, subjective well-being, and social support	135 colorectal patients undergoing post-operative chemotherapy	Randomized controlled clinical trial: TBR, telephone support (TS), and control (CON) groups (45 per group). Participants were administered the Self-Rating Depression Scale (SDS) and HAMD-17 for depression, and the Self-Rating Anxiety Scale (SAS) and HAMA for anxiety.	Post-intervention HAMD and SDS were significantly lower only for the TBR group six weeks after. TBR also reduced anxiety to the same extent like TS.
Wang et al. [26]	2011	China	To investigate the level of anxiety and depression in liver transplant (LT) recipients and analyze their impact factors	53 post-LT recipients (LT group) and 48 patients with benign end-stage liver disease (BELD group)	Cross-sectional study. SDS and the SAS were used, and the scores were compared with domestic normal values.	Anxiety and depression were greatest in the BELD group, followed by LT, and least in the domestic norms group. The impact factor was age for anxiety and monthly family per capita income for depression.
Andersen et al. [27]	2010	Norway	To assess anxiety and depressive symptoms before and after duodenal switch surgery and investigate whether the change could be explained by patient-reported physical health	50 patients undergoing duodenal switch surgery	Prospective observational study. Patients filled out the HADS questionnaire and the Short Form-36 before surgery, at one year, and at two years after surgery.	Symptoms of depression and anxiety were higher before surgery than one or two years after surgery. Physical health improved after surgery.
Donoghu et al. [28]	2003	Australia	To clarify the predictors of hysterectomy psychological outcome and illuminate possible psychological processes	60 women undergoing hysterectomy	Prospective observational study. Patients completed self-reported questionnaires for depression (Beck Depression Inventory (BDI)), anxiety STAI, personality traits, and coping at least two weeks before surgery and three months after surgery.	34% of participants reported depression before surgery and 8% three months after surgery, while 29% had anxiety before surgery and 22% after. The main risk factors for post-operative negative mood were pre-operative depression and concerns about surgical outcome.
Yang et al. [29]	2009	China	To evaluate the incidence of anxiety and depression in patients undergoing general anesthesia and analyze the relationship with peri-operative characteristics	Patients undergoing general anesthesia	Longitudinal observational study. HADS questionnaire was administered before and after surgery, and demographic and clinical data were obtained.	The pre- and post-operative rates of anxiety were 23% and 17, and for depression were 20% and 16%, respectively. Post-operative depression was predicted by female sex, pharyngeal irritation, pre-operative depression, and post-operative anxiety (vice versa).
Aykut et al. [30]	2026	Turkey	To evaluate the relationship between pre-operative anxiety, post-operative anxiety, and quality of recovery after hepatobiliary surgery	113 patients undergoing hepatobiliary surgery	Prospective observational study. STAI and State and Trait Anxiety Scale were used to score anxiety before and after surgery, and the Quality of Recovery-40 Scale was used to assess recovery.	Anxiety level was greatly reduced after surgery. Post-operative anxiety level was negatively correlated with quality of recovery and positively correlated with hospital stay and duration of anesthesia. Risk factors for anxiety included open surgery, major surgery, female sex, and low level of education.
Butt et al. [31]	2017	USA	To assess the trends and predictors of psychological outcomes of adult living donors over two years	271 adult live donors	A prospective cohort study. They were interviewed using the Primary Care Evaluation of Mental Health Disorders (PRIME-MD) once before surgery and at 3, 6, 12, and 24 months after donation.	Throughout the two years, patients presented with persistent low rates of depression (0%-3%) and anxiety (2%-3%). Predictors of mental well-being were male sex, age, relation to recipient, ambivalence and motivation to donate, and feeling that donation will make life worthier.
Wei et al. [32]	2024	China	To evaluate the effect of reminiscence therapy (RT)-engaged care (RTEC) in elderly post-operative cervical cancer patients	112 elderly post-operative cervical cancer patients with anxiety or depression	A randomized controlled study: RTEC group (56) and regular care group (56) for an intervention that lasted 12 weeks. Evaluations were performed at baseline and at the 4th, 8th, and 12th weeks using the HADS.	Depressive scores were reduced at the 8th and 12th weeks in the intervention group with respect to the control group, and anxiety scores were reduced at the 12thweek.
Ho et al. [33]	2018	Canada	To investigate the impact of post-operative complications on psychological well-being of bariatric surgery patients	365 bariatric surgery patients	Retrospective cohort. Data on post-operative complications were recorded, and data on pre-operative and one-year post-operative depression, anxiety, and quality of life (QOL) were obtained.	Compared to baseline, both groups improved in depression, anxiety, and physical QOL but not in mental QOL, which was only observed in the group without complications. The degree of improvement was even higher afterward.
Tariq and Ammar [34]	2026	Pakistan	To assess anxiety and depressive symptoms following bariatric surgery and identify associated factors	300 bariatric surgery patients	Cross-sectional study. PHQ-9 for depression and the GAD-7 for anxiety were administered at least one year after sleeve gastrectomy (SG) or gastric bypass surgery.	SG patients had lower anxiety and depressive symptoms than gastric bypass patients. Total weight loss was negatively correlated with depressive and anxiety symptoms.
Monte et al. [35]	2018	USA	To investigate the alteration in the level of depression and anxiety, and medication use after SG	51 SG patients	Longitudinal observational study. Subjects who completed SG with a diagnosis of anxiety and/or depression and were treated with medication were contacted three to six months after surgery, and the seven-point Global Impression of Change (PGIC) scale was administered.	21 were diagnosed with anxiety; symptoms improved in 12, worsened in 3, and the dosage was reduced in 5. 51 had depression; 34 improved and 4 worsened; 11 discontinued, and 4 reduced the dosage.
Azin et al. [36]	2014	Canada	To evaluate setbacks to access and compare psychological and socioeconomic characteristics between bariatric and contouring surgery and bariatric surgery alone	58 undergoing bariatric surgery alone or with contouring surgery	Cross-sectional study. Depression (Patient Health Questionnaire nine-item scale), anxiety (Generalized Anxiety Disorder seven-item scale), and QOL (Short Form-36) were assessed.	Anxiety and depressive scores were significantly higher in bariatric surgery patients than in those who underwent bariatric surgery and contouring surgery.
Neel et al. [37]	2024	Saudi Arabia	To evaluate the effect of contouring surgery on psychological state, body image, and QOL of bariatric surgery patients	227 bariatric surgery patients, with 112 undergoing body-contouring surgery	A cross-sectional study. PHQ-9 and the GAD-7 scales were used to assess depression and anxiety, respectively.	The prevalence of anxiety was 4% in the body-contouring group and 6% in the group that did not undergo body contouring. Likewise, the prevalence of depression was 6% in the body-contouring group and 8% in the other group.
Leppert et al. [38]	2007	USA	To evaluate the percentage of women scheduled for hysterectomy who still had the desire of having a child and associated mental factors	1140 premenopausal women having hysterectomy for benign indications	Longitudinal observational study. Women were interviewed before and over two years after surgery using the Profile of Mood States (POMS).	10.5% desired to have a child. Women who desired to have a child had higher levels of anxiety, depression, confusion, and anger. The difference between the two groups persisted throughout the two-year study period.
Nie [39]	2024	China	To evaluate the impacts of a web-based positive psychological nursing model on cancer-related fatigue, negative emotions, self-management efficacy, treatment compliance, and QOL among post-operative cervical cancer patients on chemotherapy	101 cervical cancer patients who underwent surgery	Control (48) received usual care, and others received the web-based positive psychological nursing model. Hamilton Anxiety Scale (HAMA), HAMD, and other diagnostic tools were used.	Anxiety and depression levels decreased in both groups after the intervention compared to the baseline. However, the scores were even lower in the intervention group than in the control group.
Simavli et al. [40]	2014	Turkey	To evaluate the effectiveness of bupivacaine-soaked spongostan in the cesarean section wound for post-operative anxiety level, early postpartum depression rate, and satisfaction	121 women undergoing elective CS under general anesthesia, American Society of Anesthesiologists physical status I-II	Randomized clinical trial. Study group (61 bupivacaine-soaked spongostan in CS wounds) and control group (60 general post-operative care). Edinburgh Postpartum Depression Scale for postpartum depression; visual analog scale for anxiety	Throughout, there was statistically significantly lower anxiety in the study group than in the control group. Postpartum depression rates were significantly lower in the study group on Days 2 and 9 than in the control group.
Wei et al. [41]	2025	China	To investigate the incidence of anxiety and depression, predictive factors, and their impact on the QOL after intestinal tumor surgery	120 patients who underwent intestinal tumor surgery	Prospective observational study. Patients were administered on the 3rd, 7th, and 30thdayafter surgery the Hamilton Depression Scale (HAMD-17) and SAS.	The depression and anxiety scores decreased significantly with time, from the 3rd to the 30th day. Hypotension during surgery and inflammatory indices were the main predictors. QOL improved.
Guglielminotti and Li [42]	2020	USA	To investigate the concept that general anesthesia during cesarean section is associated with increased odds of severe postpartum depression, hospitalization, and suicide compared with spinal anesthesia	428,204 cesarean delivery cases	Retrospective cohort. Depression was defined using an ICD-9-CM algorithm during the hospitalization for the cesarean delivery or any readmission throughout the first year after delivery.	The prevalence of postpartum depression was 2.7/1000. General anesthesia increased the odds of depression by 54% and suicidal idea by 90% compared to regional anesthesia.
Cenzer et al. [43]	2024	USA	To outline the trajectories of post-surgical depressive symptoms in elderly patients undergoing major surgery and describe the differences in characteristics	483 patients 70 years and above undergoing orthopedic, gastrointestinal, or vascular surgery	Longitudinal observational study. Patients were asked before surgery and at 6, 12, and 18 months after surgery to complete a 15-item Geriatric Depression Scale (GDS).	Generally, depressive symptoms reduced slightly in the first six months and stagnated over the subsequent six months. There were three trajectories: 10% showed persistent high depressive symptoms; 31%, persistent moderate; 59%, persistent low.
Akinsulore et al. [44]	2015	Nigeria	To evaluate the pre- and post-operative levels of anxiety and identify pre-operative predictive factors	51 patients undergoing major elective surgery (94.1% were general, orthopedic, gynecologic, and urologic surgery)	Longitudinal observational study. STAI was used to assess anxiety.	The prevalences of pre- and post-operative anxiety were 51% and 15.7%, respectively. The mean anxiety score before surgery was significantly higher than after surgery. Fear of the results of the surgery and complications were the most common reasons for pre-operative anxiety.
Sockalingam et al. [45]	2017	Canada	To investigate the feasibility, acceptability, and efficacy of a post-operative Tele-CBT intervention in improving psychological functioning and eating disorders	19 post-bariatric surgery patients	Single-arm, uncontrolled study. They received 6 Tele-CBT sessions and completed the PHQ-9 for depression and the GAD-7 for anxiety before surgery and at 6 and 12 months after surgery.	Tele-CBT resulted in a significant reduction in the mean scores of the various scales.
Cao et al. [46]	2025	China	To assess the relationship between neoadjuvant chemotherapy (NACT) and post-operative depression and anxiety	201 gastric cancer patients undergoing surgical resection	A retrospective cohort: 57 of the 201 received NACT before surgery. HADS was used to evaluate the psychological status after surgery.	The rates of depression and anxiety were significantly higher in the NACT group even after controlling for confounders (with a cumulative effect with the number of sessions).
Che et al. [47]	2020	China	To assess the impact of pre-operative information videos and brochures with regard to anesthesia on pre- and post-operative anxiety and the effect on recovery	121 women programmed for elective CS	Prospective randomized trial. Information group and control group were evaluated for anxiety with Spielberger’s STAI and salivary cortisol 24 hours pre-surgery, after video information, and two days after CS.	Watching the information video significantly reduced anxiety perioperatively, which correlated with cortisol levels.
Ay et al. [48]	2014	Turkey	To evaluate the level of anxiety in laparoscopic cholecystectomy (LAP) patients and juxtapose with that of open cholecystectomy (OP)	333 cholecystectomy patients with cholelithiasis	Cross-sectional study. Patients were asked to complete the Beck Anxiety Inventory.	Mild anxiety was observed in OP, moderate in LAP and OP, and severe predominantly in LAP. Risk factors: female, low level of education, LAP, single, and above 25 years.
Chen et al. [49]	2024	China	To investigate whether adjunctive esketamine given perioperatively for elective cesarean section could prevent postpartum depression	298 women who underwent elective cesarean delivery	Randomized controlled trial: The intervention group received esketamine after delivery, and the control group received saline. Edinburgh Postnatal Depression Scale (EPDS) was used to evaluate for depression on day 7.	The esketamine group had a markedly lower prevalence of depression than the control. However, there was no difference between the groups on day 14, 28, and 42.
Abdullah and Al-Dujaili [50]	2023	Iraq	To evaluate the level of depression in women after hysterectomy	60 women post-hysterectomy	A cross-sectional descriptive study. Patients completed the BDI.	The prevalence of no depression was 0%, mild depression was 47%, moderate depression was 33%, and severe depression was 20%
Bahri et al. [51]	2016	Iran	To examine the relation between hysterectomy and depression three months after surgery	53 women scheduled for hysterectomy	Longitudinal study. They completed the BDI before the hysterectomy and three months after the hysterectomy.	An increase in average depression score was not recorded after the hysterectomy.
Lin et al. [52]	2026	China	To investigate if repeat cesarean raises the risk of postpartum depression compared to primary cesarean	571 women scheduled for elective repeat or primary cesarean section	Prospective Cohort. Patients were assessed with the Edinburgh Postnatal Depression Scale (EPDS) at 32 weeks of gestation, 48 hours postpartum, and 6 weeks postpartum.	There was no difference in the prevalence of postpartum depression between 2 groups at 48 hours and 6 weeks after delivery.
Harms et al. [53]	2023	Germany	To determine the level of anxiety in patients undergoing primary surgery for gastrointestinal cancer	101 patients	Longitudinal study. Patients completed (GAD-7) for anxiety and (PHQ-9) for depression at t1 (outpatient) and t2 (inpatient) before surgery, and at t3 (discharge) and t4 (30 days) after surgery.	5% had severe anxiety, and 56% had mild-to-moderate anxiety. The highest level of anxiety was noted at t1, followed by t3. The t1 anxiety was highly associated with t3 and t4, and women were more likely to have anxiety.
Tsai et al. [54]	2021	Tiawan	To explore the relationship between depressive disorders and cholecystectomy in patients with cholelithiasis	6755 patients with cholelithiasis	Retrospective cohort. Patients were followed up for two years to identify those who were diagnosed with depression.	3.51% of cholecystectomy patients and 2.36% of patients not operated on developed depression. After stratification, depression was associated with cholecystectomy in women.
Sockalingam et al. [55]	2022	Canada	To determine the effectiveness of telephone-based cognitive behavioral therapy (Tele-CBT) in ameliorating anxiety, depression, and eating disorders	81 patients 12 to 14 months post-surgery	Randomized controlled trial. Regular care (control) and Tele-CBT (intervention). Patients completed the GAD-7 and PHQ-9 at baseline, immediately after intervention, and three months after the intervention.	Anxiety and depressive follow-up scores after the intervention decreased significantly in the intervention group compared to the control group and baseline.
Zora and Gültekin [56]	2026	Turkey	To investigate the association between post-operative anxiety and pre-operative waiting time after information disclosure	235 adult laparoscopic cholecystectomy patients	Prospective observational study. Information was disclosed 24 hours before surgery (late) or 7 days before (early), and the State-Trait Anxiety Inventory-State form (STAI-State) was completed 24 hours after surgery.	The early information disclosure group had higher anxiety scores than the late disclosure group.
Hernández-Muñoz et al. [57]	2025	Mexico	To investigate the risk factors of positive postpartum depression screening in elective cesarean section (ECS) patients	123 women undergoing ECS	An analytical cross-sectional study. Edinburgh Postpartum Depression Scale (EPDS) was completed 72 hours after ECS.	32.5% of women had a positive PPD screening. Multiparity, 30 years and above, history of miscarriages, unplanned pregnancy, and maternal and neonatal complications were identified as risk factors.
Hepp et al. [58]	2018	Germany	To evaluate the effect of music during cesarean section on expectant mothers’ anxiety and stress	304 patients undergoing CS	Randomized controlled trial. The study group received music in addition to regular care during the CS under regional anesthesia. STAI was completed 7 to 14 days before surgery. STAI and objective measurements (salivary cortisol/amylase, heart rate, and blood pressure) were obtained before and 2 hours after surgery. Objective measurements were also done during surgery.	Anxiety levels and objective measurements of anxiety were significantly lower in the intervention compared to the control at the end of the intervention and 2 hours after the intervention.
Dekel et al. [59]	2019	USA	To determine whether maternal mental well-being is affected by mode of delivery	685 women had a live birth within the last six months	Retrospective cohort. Brief Symptom Inventory (BSI) was completed to evaluate nine symptom clusters.	Instrumental delivery and CS had higher anxiety, depressive, somatization, and obsessive symptoms than natural vaginal delivery. Unplanned CS, but not vaginal instrumental delivery, was associated with high PTSD symptoms.
Phetprapasri et al. [60]	2024	Thailand	To investigate whether providing informative CS videos affects maternal anxiety	178 women undergoing CS	Randomized controlled trial. The intervention group benefited from an additional five-minute video before surgery. STAI was completed on the day of the CS and the day after.	The intervention group had a significantly lower post-operative anxiety score compared to the control, despite almost similar scores before surgery.
Pinar et al. [61]	2011	Turkey	To determine whether pre-operative instructions reduce the level of post-operative anxiety	60 patients undergoing gynecologic surgeries	Randomized controlled trial. The study group was administered systematic pre-operative instruction in addition to regular care. STAI was used after the surgery.	Study group demonstrated a lower anxiety score than the control.
Belt et al. [62]	2025	USA	To assess the effect of lower extremity weight-bearing status on post-operative development of psychiatric disorders	14,820 patients undergoing either Open reduction internal fixation (ORIF) or intramedullary nailing (IMN): (7,410 patients per cohort)	Prospective cohorts, with early weight bearing after IMN on one arm and late weight bearing after ORIF on the other arm. Excluded were patients with a previous psychiatric diagnosis. Main outcome was the onset of psychiatric disorder or substance use disorder.	Early (1-7 days): no difference. Intermediate (7 days-2 months): IMN was associated with a lower incidence of depression. Long-term (more than two years): IMN was associated with a lower rate of opioid use disorder.
Ouaz et al. [63]	2026	Tunisia	To assess the impact of virtual reality on perioperative anxiety in regional anesthesia	72 orthopedic surgery patients	Randomized controlled trial: 32 patients in the virtual reality (VR) and non-VR groups. STAI-6 was used for anxiety, and the need for midazolam was noted.	With no difference at the beginning, VR reduced the need and quantity of anxiolytic (61.7% in the VR- group vs. 5.8% in the VR+ group).
Tsapakis et al. [64]	2009	United Kingdom	To assess the impact of orthopedic day surgery on mood	148 patients between 18 and 65 years old, ASA 1, requiring general anesthesia	Prospective cohort. Beck Anxiety Inventory (BAI), BDI, and the short form 36 (SF-36) for medical outcome were administered pre- and post-operatively.	Variation in presurgical mood correlated with post-surgical mood. Mood symptoms aggravated over time after surgery.
Nickinson et al. [65]	2009	United Kingdom	To study the presence and rate of post-surgical anxiety and depression	56 undergoing hip or knee arthroplasty	Prospective observational study. Patients were allowed to fill in the HADS questionnaire a day before surgery, every day after surgery up to and including the day of discharge.	25% became anxious before surgery. 50% were depressed at one point after surgery. Factors associated with depression: female, age, and previous history of arthroplasty.
Suroto et al. [66]	2021	Indonesia	To assess the relationship between disability and pain to post-traumatic stress disorder, anxiety, and depression in patients with post-operative BPI	41 post-operative brachial plexus injury patients	Cross-sectional study. Pain was assessed with the visual analog (VAS) score, disability with the Disabilities of the Arm, Shoulder, and Hand (DASH) score, and mental disorder with the Mini International Psychiatry Interview (MINI).	10 people had major depression, 2 had anxiety, and another 2 had PTSD. DASH scores were associated with depression.
Kratz et al. [67]	2010	USA	To compare psychological, pain, and social variables over the first 12 months post-amputation between traumatic and non-traumatic groups.	111 patients who recently lost their limbs	Longitudinal observational study. Post-traumatic Stress Disorder Checklist, Patient Health Questionnaire depression module, and other scales were completed initially, 1-, 6-, and 12 months post-amputation.	Outcomes did not differ between the two groups. Both groups showed a progressive increase in post-traumatic stress disorder symptoms over time. The non-traumatic group was much older.
Pan et al. [68]	2010	China	To assess the effect of music as a therapy for peri-operative depression and anxiety, and satisfaction after surgery for distal radius fractures	186 patients, 65 years and above	Retrospective cohort: Patients with standard care and those who received music therapy in addition. HADS and other scores were completed pre-surgery, 24 hours after surgery, discharge, 2 weeks, and 6 weeks after surgery.	The group that received music therapy had lower levels of depression and anxiety, better sleep and satisfaction, and a reduced number of days of hospitalization.
Emani et al. [69]	2026	USA	To evaluate the relation between the psychological and physical aspects of the brachial plexus injury (BPI)	44 traumatic BPI	Retrospective cohort. The Posttraumatic Stress Disorder (PTSD) Checklist for Diagnostic and Statistical Manual of Mental Disorders: Fifth Edition (PCL-5) and others were used to interview the patients.	18% met the criteria for PTSD.
Jiang et al. [70]	2016	China	To test the effect of intraoperative administration of ketamine on post-operative depressive mood in orthopedic surgery patients	120 patients undergoing elective orthopedic surgery	Randomized, double-blind, controlled trial. Ketamine group and control group. The intervention group was given ketamine as part of the induction drugs, and the control group was excluded. PHQ-9 score for depression was assessed a day before surgery, 1 day, and 5 days after surgery. Serum brain-derived neurotrophic factor (BDNF) was drawn at the same time.	There was no difference in pre-operative characteristics between the two groups, including PHQ-9 scores. There was a significant reduction in the post-operative depressive score of the intervention group when compared to either the pre-surgical intervention group or post-surgical control (which remained the same as the pre-surgical).
Shi et al. [71]	2021	China	To compare the impact of the 3 different total hip arthroplasty (THA) approaches on post-operative depression and anxiety	95 THA	Randomized controlled trial. HADS was completed before surgery and three months after surgery.	Normal lateral approach (NLA) had higher post-operative scores than the direct anterior (DAA) and the orthopädische chirurgie München (OCM) approaches.
Demnati et al. [72]	2024	Morocco	To assess the psychological effect of peri-implant fracture (PIF)	136 patients with PIFs	Cross-sectional study. HADS and Perceived Stress Scale (PSS) were completed.	Patients with PIFs had anxiety, depressive, and post-traumatic stress disorder scores above those of the general population.
Kim et al. [73]	2023	South Korea	To evaluate the correlation between anxiety and levels of Functional Near-infrared Spectroscopy (fNIRS) in patients above 65 years undergoing joint replacement surgery	60 patients undergoing joint replacement surgery	Randomized trial. Patients were partitioned into three groups: normal saline (control), dexmedetomidine at 0.2 μg/kg/h (group 2), and 0.5 μg/kg/h (group 3). The fNIRS and STAI were completed during the pre-anesthetic phase, spinal anesthetic phase, and after either normal saline or dexmedetomidine.	fNIRS power of bandwidth was highly correlated with the STAI score.
Seid Tegegne and Fentie Alle [74]	2022	Ethiopia	To evaluate the extent and factors associated with post-operative depression among orthopedic surgery patients	443 adults undergoing orthopedic surgery	Cross-sectional study. PHQ-9 score was obtained before surgery as baseline and on the day of discharge.	The prevalence of post-operative depression was 61.8%. Substance use, female sex, underweight, history of anxiety, being a farmer, open fracture, and lack of social support
Orovou et al. [75]	2022	Greece	To investigate the relationship between previous traumatic life events and PTSD after cesarean delivery	469 post CS women	Prospective study. Post-traumatic stress checklist was used.	Prevalence of PTSD was 25.97%. Emergency CS and preterm birth were risk factors.
Chandrasekaran et al. [76]	2018	Canada	Investigated whether postpartum anemia (PPA) is an independent risk factor for new-onset postpartum depression (PPD)	103 post-elective CS women	Cross-sectional study. Edinburgh Postnatal Depression Scale was used.	17% had depression at 6 weeks. Anemia was not an independent risk factor.
Doke et al. [77]	2021	India	Compared PPD at six weeks between vagina delivery and cesarean delivery women	2831 women after vaginal and cesarean birth	Cross-sectional study. Edinburgh Postnatal Depression Scale was used.	Prevalence in CS women was higher. Age below 25 was a risk factor.
Garthus-Niegel S et al. [78]	2014	Norway	Assessed whether a mismatch between actual and desired mode of delivery increases risk of PTSD	1,700 women scheduled for CS	Questionnaires were administered at 17 and 32 weeks and from 8 weeks postpartum.	PTSD was more frequent in women who preferred CS but delivered vaginally.
Lin et al. [79]	2022	China	Investigated risk factors for PPD after CS	590 women who underwent elective CS	A prospective cohort study. EPDS was used.	Pain and prenatal depression were risk factors.
Grisbrook et al. [80]	2022	Canada	Assessed the association between CS type and PPD and PTSD	354 who underwent CS	EPDS and the Psychiatric Diagnostic Screening Questionnaire (PDSQ) were administered three months after CS.	Emergency CS was associated with PPD indirectly through PTSD symptoms.
Baba et al. [81]	2023	Japan	Investigated association between mode of delivery and risks of PPD	89,954 mothers	Prospective study. EPDS was administered at one and six months postpartum.	Depression was more prevalent in CS delivery than CS at one month but not at six.
Kuo et al. [82]	2014	Taiwan	To describe the trajectories of depression and anxiety after CS	Childbearing women (N=139) who underwent a CS	Prospective longitudinal study	Similar trajectories were observed between depression and anxiety.
Sun et al. [83]	2020	China	To assess if epidural anesthesia during labor reduces the risk of PPD after CS	Analgesia group (n=263) or non-analgesia group (n=160)	Prospective cohort. EPDS was administered at 48 hours and 42 days after CS.	Prevalence of depression was lower in the analgesic group throughout.
Wan et al. [84]	2024	China	Investigated the effects of subclinical doses of esketamine on PPD and pain after CS	150 pregnant women undergoing elective CS	Randomized, double-blind, placebo-controlled trial. EPDS was administered at 3rd, 7th, and 14th day.	There was no significant difference.
Liu et al. [85]	2022	Taiwan	To study whether mode of birth is related to PPD	32,729 women who delivered through CS or vaginally	Prospective study. PPD was administered at 6 and 12 months after birth.	CS was associated with a higher rate of PPD.
Xu et al. [86]	2025	China	To study whether mode of birth is associated with PTSD	759 postpartum women	Prospective cohort. Post-traumatic Stress Disorder Checklist-Civilian assessed at 42 weeks.	The prevalence of postpartum PTSD was 17.55% in CS women and 8.18% in natural birth women.
Zhang et al. [87]	2025	China	To investigate the effect of esketamine intravenous analgesic pump on PDD and pain	168 CS delivery women	Retrospective cohort. EPDS was used.	EPDS scores were lower in the esketamine group than in the non-esketamine group.
Agarwal et al. [88]	2023	India	To study whether mode of birth is related to PPD	121 in the normal delivery (ND) group and 121 in the CS group	Prospective cohort. EPDS was used.	Prevalence of depression was higher in CS (34.71%) than in the ND group (19.83%).
Özalp et al. [89]	2026	Turkey	To compare the rates of PPD between women undergoing CS after prior vaginal delivery and women undergoing vaginal delivery	120 women post-cesarean delivery and 120 women post-vaginal delivery	Retrospective observational study. EPDS was used.	Depression was more prevalent after CS (26.7%) than natural birth (10%), and there was no difference between elective and emergency CS.
Theunissen et al. [90]	2017	Netherlands	To determine predictive factors of depression and wellbeing after hysterectomy	419 women undergoing hysterectomy for benign indication	Prospective multicenter cohort study	Post-operative depression improved over time.
Senturk et al. [91]	2017	Turkey	To study PPD after emergency peripartum hysterectomy and associated factors	41 patients (17 non-hysterectomy and 24hysterectomy patients)	Cross-sectional controlled study. EPDS was used.	Depression was higher in the hysterectomy group.
Wang et al. [92]	2020	China	To study the effects of various doses of S-ketamine on depression and pain management of cervical carcinoma patients with mild/moderate depression	417 cervical carcinoma patients who received laparoscopic modified radical hysterectomy	Randomized, double-blind, controlled study. Four groups: 1) the control group, 2) the racemic ketamine group, 3) the high-dose S-ketamine group, and 4) the low-dose S-ketamine group. HAMD and VAS were used.	The high-dose ketamine group showed lower levels of pain and depression on the 1st and 3rd day than other groups. At one week, there was no difference between the four groups.
Spüntrup et al. [93]	2022	Germany	To investigate the effect of persistent bleeding on anxious and depressive symptoms	311 patients: conical excision (n=163), and straight cervical resection (n=148)	Prospective cohort. German version of HADS, HADS-D were used,	Both procedures reduced depression and anxiety at 3 and 12 months after surgery to the same extent.
Ghotbizadeh et al. [94]	2021	Iranian	To study the prevalence of depression and sexual dysfunction after hysterectomy	Fifty-two patients with a cesarean hysterectomy and 52 with cesarean section	Case-control study. Beck Depression Index was used to evaluate depression three to six months after surgery,	There was a difference in the level of depression between two groups (average of two groups), although there was a difference in the prevalence of mild to moderate.
Kim et al. [95]	2021	South Korea	To investigate the relationship between depression and oophorectomy	oophorectomy (n=36,284) and hysterectomy (n=25,415)	Self-controlled case series design. National Health Insurance Claims Database was used to obtain data,	There was increased depression before and after surgery for both groups. This persisted up to one year after surgery.
Oberlin et al. [96]	2026	USA	To assess the prevalence of PTSD, anxiety, and depression in patients who underwent hysterectomy for placenta accreta	61 post-hysterectomy patients	Cross-sectional study. National Stressful Events Survey short scale for symptoms of PTSD, the General Anxiety Disorder scale, and the Patient Health Questionnaire were administered.	21.3% were positive for PTSD, 16.4% for anxiety, and 13.1% for depression.
Ahdi Derav et al. [97]	2023	Iran	To evaluate the effectiveness of group cognitive-behavioral therapy (GCBT) in reducing anxiety and depression in women who underwent hysterectomy for uterine cancer.	26 post-hysterectomy women with uterine cancer	Experimental, pretest-post-test study. Beck Anxiety (BAI) and Beck Depression-second version (BDI-II) questionnaires were completed by patients.	GCBT significantly reduced anxiety and depression, while depression and anxiety remained the same pre- and post-intervention in the control group.
Wang et al. [98]	2007	China	To assess the relationships among anxiety, depression, coping strategies, and demographic characteristics of post-hysterectomy	105 post-hysterectomy women	Cross-sectional study. Zung SAS and Zung SDS were completed.	Prevalence of anxiety was 1.9%, and depression was 4.8% within the last two days before discharge.
Vandyk et al. [99]	2011	Canada	To investigate factors associated with depressive symptoms before and after hysterectomy	384 women undergoing hysterectomy	Cross-sectional study. Patients were administered the questionnaire on admission and six months after surgery.	Prevalence of depression was 36% before and 22% after surgery. Incidence was 6%, and 15% had persistent depression at both moments.
Duggal et al. [100]	2014	UK	Investigated the synergistic effect of depression and physical stress on the immune system	101 hip fracture patients (81 female) and 43 healthy age-matched controls (26 female)	Case-control study. HADS was completed six weeks after the intervention.	38 out of 101 fracture cases had depression. Altered T-cell phenotype and increased pro-inflammatory function were noted.
Kornfield et al. [101]	2017	US	To investigate if fear of falling after hip fracture is associated with increased risk of PTSD	456 individuals aged 60 and above admitted for hip fracture surgery	Cross-sectional study. Montgomery-Åsberg Depression Rating Scale (MADRS) was administered on admission and 12 weeks after surgery.	Hip fracture was not associated with full PTSD. However, hip fracture surgery was associated with partial PTSD.
Cai et al. [102]	2024	China	To evaluate whether adjunctive esketamine in patient-controlled intravenous analgesia (PCIA) could improve depressive symptoms in elderly patients undergoing hip fracture surgery	90 patients, aged ≥65 years, undergoing hip fracture surgery	A double-blind, randomized controlled clinical trial. 0.5 mg/kg of esketamine as a PCIA adjuvant for 48 h, and GDS-15 was administered sequentially.	The prevalence of depression in the esketamine group (28.6%) was lower than in the control group (53.5%). Similarly, the esketamine group had higher levels of brain-derived neurotrophic factor (BDNF) and 5-hydroxytryptamine (5-HT).
Cristancho et al. [103]	2016	USA	To describe the trajectories of depressive symptoms arising after hip fracture and examine their relationship with functional outcomes and walking ability	482 inpatients, aged ≥60 years undergoing fracture repair	Prospective study. Montgomery-Asberg Depression Rating Scale and Functional Recovery Score were administered for 52 weeks.	The majority maintained a low level of depression throughout (51%); 38.1% increased after surgery but later returned to original levels; 10% had persistent high levels.
Schwartz et al. [104]	2021	USA	To study the risk factors for post-operative depression after total knee arthroplasty (TKA) and assess its association with post-operative complications	196,728 unique TKA patients	Case-control study. Patients diagnosed with depression within a year were cases, and others were controls.	Incidence of depression was 2.7%. Age <54 years old, multiple comorbidities, pre-operative anxiety disorder, drug, alcohol, and/or tobacco use, female gender, and opioid use before TKA were associated factors.
Lenze et al. [105]	2007	USA	To investigate predictors of onset of major depressive disorder (MDD) and of depressive symptoms after hip fracture surgery	126 subjects undergoing hip fracture surgery	Longitudinal study. Hamilton Rating Scale for Depression (HAMD) was administered on discharge, two weeks, and six months later.	Incidence of major depression was 14.3% (18/126), 11 developed at the end of the hospitalization, and 7 between the 2nd and 10th week.
Burns et al. [106]	2007	UK	To assess the effect of a psychiatric intervention in treating depression (treatment study) and the effect of a psychological treatment in preventing depression (prevention study) following hip fracture	293 elderly patients undergoing hip fracture repair	A randomized controlled trial. GDS and HADS for mood were completed.	Depression decreased in the treatment group but not in the prevention group.

Characteristics of the Included Studies

Publication trends and study designs: Of the 86 studies, the most common were prospective observational studies (n=22), followed by randomized controlled trials (n=21), cross-sectional studies (n=18), prospective cohort studies (n=11), retrospective cohort studies (n=8), case-control studies (3), a retrospective, longitudinal, mirror-image study (n=1), a self-controlled case series (1), and an uncontrolled single arm clinical trial (n=1).

**Figure 2:**
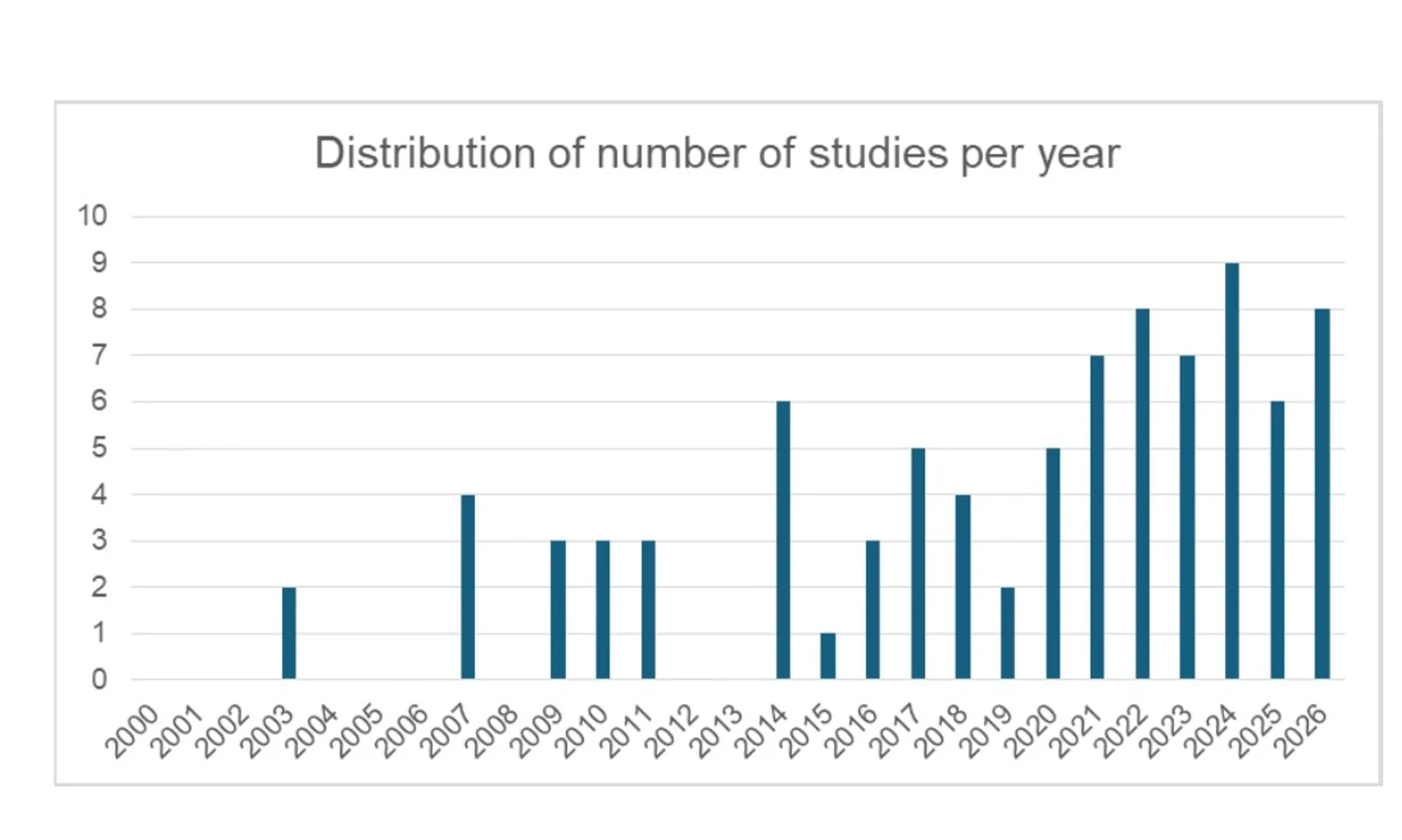
Number of studies published per year

Overall, 75.6% (65) of the studies were published from 2015, showing an increasing research interest in the impact of surgery on mental health. There was wide variation in sample sizes, ranging from 19 to 4,228,204 participants [42,45]. Figure 2 shows the number of studies published per year.

Characteristics of the Population

All studies excluded pediatric patients, some excluded individuals with pre-existing mental health conditions, severe medical comorbidities, or older adults aged ≥65 years, whereas specific orthopedic studies intentionally focused on geriatric populations [43,62,68,73]. Conversely, certain studies enrolled patients with pre-existing depression and anxiety to analyze pre- and post-operative symptom trends [35,78]. Women constituted the entire cohort in gynecological studies, whereas both genders were well represented across the orthopedic and gastrointestinal surgical categories.

Most studies were conducted in high- and upper-middle-income nations, with China being the most highly represented (n=21), followed by the United States (n=14), Turkey (n=7), and Canada (n=7). Notably, limited evidence originated from low-income countries, including three studies from sub-Saharan Africa, two from Nigeria, and one from Ethiopia [24,44]. We found no study in South America. Thus, Asia accounted for most of the literature, followed by North America, Europe, and Africa.

Surgical Specialties

The included studies were classified into four main surgical categories: gynecological and obstetrical (n=41, including hysterectomies, cesarean sections, and oophorectomies), digestive (n=22, including bariatric surgeries, cholecystectomies, and gastrointestinal oncological resections), orthopedic (n=20, including knee arthroplasty, osteosynthesis, and total hip arthroplasty), and cross-specialty (n=3).

Assessment Tools Used

Most studies assessed mental health using self-reported, standardized questionnaires to estimate the prevalence and severity of depression. However, few relied on physician assessments coded as the diagnosis using the International Classification of Diseases (ICD)-9-CM code, which records clinical diagnoses for insurance reimbursement. Among the instruments evaluating post-operative depression, the Hospital Anxiety and Depression Scale (HADS) was the most frequently used, followed by the Patient Health Questionnaire-9 (PHQ-9). The HADS features two 7-item subscales: HADS-D for depression and HADS-A for anxiety, with each item scored from 0 to 3, yielding a total subscale range of 0 to 21. Scores ≥8 indicate possible depression, while scores ≥11 signify severe depressive symptoms.

The PHQ-9 and the Generalized Anxiety Disorder-7 (GAD-7) scales were used frequently. Both score individual items from 0 to 3, but the 9-item PHQ-9 ranges from 0 to 27, whereas the 7-item GAD-7 ranges from 0 to 21. Since both scales categorize symptoms into minimal, mild, moderate, and severe bands, they are frequently paired. For specific populations, the Edinburgh Postnatal Depression Scale (EPDS) was used exclusively for postpartum depression (though a few postpartum studies used ICD-9-CM codes), while the Geriatric Depression Scale was used solely for elderly patients. Finally, PTSD symptoms were primarily evaluated using the PTSD Checklist for the Diagnostic and Statistical Manual of Mental Disorders, fifth edition (PCL-5), with scores ranging from 0 to 80. The PCL-5 tracks symptom onset, evolution, and severity, with a 5- to 10-point shift indicating reliable change and a 10- to 20-point shift representing clinically significant change. The complete list of the various assessments has been provided in the appendix.

Distribution of Evidence

Orthopedic surgeries: There was a wide variation in the estimated prevalence and incidence of post-operative depression, with elevated rates reported among geriatric populations, individuals with hip fractures, and patients experiencing surgical complications. The prevalence of depressive symptoms ranged from 10% to 50% [65,66,100,103,105]; however, this prevalence exceeded 50% in some studies, including an investigation conducted during the COVID-19 pandemic that documented a prevalence of 61.8% [74,102]. The incidence of post-operative anxiety symptoms ranged from 4% to 61.7%. The incidence of PTSD symptoms was generally <18%; however, a higher susceptibility was noted among amputees [66,69]. Generally, symptoms of anxiety and depression were ameliorated after surgical intervention involving the successful restoration of mobility [62,103,105].

Gynecologic surgeries: Conflicting information was reported regarding the impact of hysterectomies on post-operative depression; while certain studies reported higher rates of depression [38,50,90], others highlighted an improvement in depression and anxiety [28,32,93,99]. The prevalence of post-operative postpartum depression was 30%, a rate exceeding that of women undergoing vaginal delivery; however, no significant difference was noted between women undergoing primary cesarean sections versus repeat procedures [76,86,88,89]. Cesarean delivery, particularly in emergency situations, resulted in a higher severity of PTSD symptoms than vaginal delivery [59,75,80]. Notably, interventions such as informative educational videos regarding cesarean sections reduced pre- and post-operative anxiety, while the administration of esketamine for induction, the playing of music during cesarean delivery, the application of bupivacaine-soaked Spongostan on surgical wounds, and comprehensive pre-operative instructions were associated with reduced severity and lower prevalence of post-cesarean section depression [40,47,49,58,60,84,87]. Conversely, the administration of general anesthesia was associated with a higher prevalence of depressive symptoms than regional anesthesia [42,83].

Digestive surgeries: Mental health outcomes were generally mixed among patients undergoing digestive surgery, with certain procedures demonstrating higher anxiety and depressive symptom prevalences than others. Following bariatric surgery, the prevalence of depression and anxiety was generally <10%, and post-operative depression and anxiety levels decreased over time [23,27,33,35,37]. Conversely, a study reported significantly higher levels of depression in patients undergoing cholecystectomy for gallstone disease than in untreated controls [54]. Furthermore, laparoscopic cholecystectomy was associated with higher levels of post-operative anxiety than open surgery, particularly when patients were informed of the procedure well in advance [48,56]. Finally, interventions for gastrointestinal cancers revealed high levels of persistent post-operative depression and anxiety [21,46].

Risk and Protective Factors for Mental Health Conditions

Depression and anxiety were more likely to manifest in women, illicit drug users, underweight individuals, patients experiencing pain, geriatric populations, and patients with a history of similar surgical interventions. Furthermore, individuals undergoing major, open, complicated, or oncological surgeries under general anesthesia, and those with low per capita income, lower educational attainment, and minimal or absent social support networks were more vulnerable to depression and anxiety. Conversely, pre-operative counseling, psychotherapy, minimally invasive procedures, and corrective or weight-loss surgeries were protective factors against depression and anxiety. Regarding PTSD, patients undergoing amputations or cesarean deliveries and those experiencing peri-implant fractures demonstrated a significantly higher susceptibility. Finally, the primary risk factors for postpartum depression included multiparity, maternal age ≥30 years, unplanned pregnancy, a history of miscarriages, and the presence of maternal or neonatal postpartum complications [26,29,30,31,57,65,77,79,80,83].

Emerging Diagnostic and Therapeutic Approaches

According to randomized controlled trials, ketamine effectively alleviated post-operative depressive symptoms, while intraoperative music therapy significantly mitigated post-surgical anxiety and depression. Additionally, virtual reality interventions and the pre-operative provision of informative educational videos substantially reduced anxiety levels. In bariatric surgery, tele-behavioral cognitive therapy successfully lowered the severity of anxiety and depression. Similarly, the application of bupivacaine-soaked Spongostan within cesarean section wounds reduced the risk of postpartum anxiety and depression. Finally, functional near-infrared spectroscopy (fNIRS) represented an objective modality for quantifying anxiety severity in geriatric populations.

Evidence Gaps

There was a wide variation in assessment tools, follow-up durations, and outcome interpretations, which limits the synthesis, comparison, and mapping of results. For example, studies did not unanimously report the incidence (new-onset symptoms), prevalence, evolution of symptoms after surgery compared to pre-operative baseline, and trajectories of symptoms over time following surgery. Few studies originated from low-income countries. Some studies failed to compare outcomes across different surgical specialties, and multinational investigations were not conducted. Furthermore, no experimental studies have been conducted to elucidate the underlying biological mechanisms driving post-surgical mental health outcomes. There were also no standardized assessment scales specifically validated for post-operative mental health outcomes. Finally, urological surgery-specific studies were unrepresented, and studies focusing on PTSD were scarce.

Discussion

We conducted this scoping review to map the breadth and depth of evidence regarding post-operative mental health following abdominal and orthopedic surgeries. Specifically, we focused on post-operative depression, post-operative anxiety, and PTSD, which are ranked among the most prevalent psychological complications following surgical procedures [2]. Of 29,536 records retrieved during our search, 86 studies met the inclusion criteria. Prospective observational studies were the most frequent (n=22), followed by randomized controlled trials (n=21) and cross-sectional studies (n=18). Most studies originated from China (n=21) and the USA (n=14). Notably, there was a stark paucity of data from low- and middle-income countries (LMICs), with South America being the least represented continent. The predominance of publications from China and the USA underscores their robust engagement in mental health research. This output aligns with the 2024 Nature Index, which ranked these two nations as global leaders in scientific research [107].

Among studies on abdominal surgery, one-third focused on gastrointestinal interventions, with particular emphasis on oncological resections and bariatric procedures. This concentration mirrors the escalating global burden of non-communicable diseases such as obesity and malignancy, which are currently two of the most critical public health concerns [108]. Investigations tracking patients undergoing bariatric surgery revealed that mental health outcomes, specifically symptoms of depression and anxiety, were exacerbated during the immediate perioperative phase but subsequently improved after surgery [22,27,33-35]. This psychological recovery was significantly more pronounced when bariatric interventions were combined with body contouring surgery [36,37] or when sleeve gastrectomy was preferentially performed over gastric bypass [34]. This initial post-operative symptom exacerbation may be driven by the immediate impact of surgery as a profound psychological stressor, or the development of early post-surgical complications, as reported by a study on sleeve gastrectomy [33]. The subsequent long-term improvement in mental health following bariatric interventions can be elucidated by substantial weight loss, an enhanced perception of body image, routine post-operative psychotherapeutic support, and elevated self-esteem, a trajectory further corroborated by the superior mental health outcomes documented following body contouring procedures.

Similarly, the acute post-operative phase in orthopedic patients was characterized by elevated rates and peaking levels of depression compared to that at the pre-surgical baseline [64,65,74]. Orthopedic procedures were the third most highly represented surgical category in this review. However, the lower occurrence of orthopedic surgery in our review is attributable to the fact that spine surgery studies and investigations evaluating HRQOL, though substantial in volume, were excluded from our study. HRQOL is a multidimensional, subjective and objective evaluation of the physical and psychological well-being of a patient experiencing a specific clinical condition, with the physical domain inherently predominating [109]. The six core dimensions of HRQOL include physical functioning, role limitations, bodily pain, mental health, vitality, and social functioning [110]. Since rehabilitation and restoration of physical function are of paramount importance in orthopedics, a vast body of literature focusing on HRQOL was retrieved and excluded from this study due to the complexity of mapping such extensive data within our specific scoping framework.

The findings regarding depressive outcomes in patients who underwent a hysterectomy were conflicting. Among the eight studies comparing pre- and post-operative depression after hysterectomy, four revealed an improvement in depression levels [28,32,93,99], one revealed no significant difference between pre-operative and post-operative depression [51], and three reported worsened post-surgical depression levels [38,50,90]. Although clinically indicated hysterectomy can be beneficial, such as in cases of malignancy, endometriosis, or pyometra [111], it can also be detrimental to psychological well-being, particularly for women who are sexually active, still menstruating, or desiring children. Notably, instrumental and cesarean deliveries were associated with higher levels of anxiety, depression, and PTSD compared to vaginal deliveries [59]. This disparity may be elucidated by the fact that operative deliveries are more invasive, more costly, and frequently performed in emergency situations without adequate pre-operative psychological preparation. Furthermore, these interventions are often associated with higher risks of maternal and fetal morbidity and mortality. Conversely, repeat cesarean sections were not associated with higher levels of depression when compared to primary cesarean deliveries [52]. This specific outcome may be attributable to the psychological phenomenon of desensitization.

Across specialties, female sex, advanced age, a lack of social support, low socioeconomic status, and a history of psychiatric conditions emerged as prominent factors influencing psychological disorders [57,65]. The studies that documented the highest prevalence rates of depression and anxiety were conducted in Ethiopia and Tunisia, respectively, among orthopedic patients undergoing arthroplasty [63,74]. Findings within LMICs among arthroplasty cohorts who are predominantly geriatric patients underscore the reality that age and lower socioeconomic status significantly amplify the risk of psychiatric morbidity. Geriatric patients frequently present with multiple medical comorbidities, such as neurodegenerative diseases, and exhibit reduced resilience to surgical stress alongside higher rates of post-operative complications. This multifaceted vulnerability elucidates the high rates of depression observed among older adult patients undergoing total hip arthroplasty.

A common finding across surgical specialties was that specific operative approaches for the same underlying condition resulted in significantly superior psychological outcomes. Examples included sleeve gastrectomy over Roux-en-Y gastric bypass [34], bariatric surgery combined with body contouring over bariatric surgery alone [36,37], open surgery over laparoscopic cholecystectomy [48], conservative management over surgical intervention for cholelithiasis [54], the direct anterior approach and the Orthopädische Chirurgie München techniques over the conventional lateral approach [71], intramedullary nailing with early weight-bearing over open reduction and internal fixation [62], spinal anesthesia for cesarean delivery over general anesthesia [42,83], and vaginal delivery over cesarean section [59]. Consequently, to ensure holistic patient care, it is essential to carry out large-scale studies to investigate these findings, which will inform decisions regarding balancing surgical indications and clinical techniques with potential post-operative mental health outcomes, particularly in patients presenting with identifiable risk factors.

One of the key findings of this review was the marked heterogeneity across the assessment tools, reported prevalence rates of psychological outcomes, and chronological timing of post-surgical mental health evaluations. Most investigations relied heavily on self-reported screening inventories, whereas only a limited number of studies integrated healthcare provider assessment instruments or prescription medication databases. Furthermore, a nominal subset of studies implemented objective physiological measures, such as salivary cortisol levels and fNIRS, to quantify anxiety severity. The EPDS was the only clinical instrument consistently and unanimously deployed across all studies within a specific domain to evaluate postpartum depression. However, the diagnostic threshold used to define major depression varied significantly between investigations, with individual studies adopting cut-off scores of 9, 10, 11, 12, or 13 and above. This diagnostic variability ultimately complicates a precise and unified estimation of the true prevalence of post-operative postpartum depression.

Implications

This study provides critical insights into the impact of surgical interventions on the mental health of patients undergoing abdominal and orthopedic procedures. The findings highlight gaps in the continuum of care, particularly regarding common emergency and elective procedures such as appendectomy, intestinal obstruction resection, gastrointestinal perforation repair, urological surgeries, laparotomy for ectopic pregnancy, and operations for ovarian cyst torsion or rupture. Therefore, targeted investigations evaluating the post-operative mental health needs of patients undergoing these prevalent abdominal and orthopedic surgeries are urgently needed. Furthermore, given that specific surgical techniques might yield superior psychological outcomes and that mental health significantly influences surgical recovery, it is essential to conduct large-scale comparative studies. These investigations should evaluate the outcomes of different operative approaches while strictly controlling for baseline psychological status, as observed clinical differences may be driven by confounding variables or effect modifiers. Although certain procedures are inherently more traumatic than others, future research must evaluate PTSD among patients undergoing abdominal surgeries other than cesarean sections, and orthopedic interventions beyond amputations and brachial plexus injuries. Finally, this review underscores the necessity for large-scale, multinational studies to adequately investigate the efficacy of perioperative interventions designed to mitigate anxiety and depression, including the administration of certain intraoperative N-methyl-D-aspartate (NMDA) receptor antagonists to attenuate post-operative depression, the application of local anesthetic-impregnated dressings for wound care, and the implementation of virtual reality gaming during spinal anesthesia.

Our findings inform surgeons of the critical need to incorporate patients' psychological well-being when choosing between surgical or anesthesiologic techniques, managing surgical complications, determining the indication to operate, and addressing expected delays in patient recovery. This integration is crucial because post-operative complications, alongside specific surgical and anesthesiologic approaches, could directly influence mental health, which could delay recovery and detrimentally affect overall surgical outcomes. Furthermore, this study underscores the need for a structured, multidisciplinary approach involving surgeons, anesthetists, psychiatrists, and psychologists across the pre-, intra-, and post-operative phases to optimize clinical and psychological outcomes, thereby ensuring holistic patient care.

This review also underscores the need for patient-centered care and thorough pre-operative preparation. Before surgical intervention, it is essential to identify individuals presenting with prominent risk factors for psychological distress, determine the optimal volume and timing of procedural information to be shared, and provide patients with targeted educational materials such as informative brochures and videos. Furthermore, psychological therapies and careful selection of optimal surgical and anesthetic techniques should be prioritized during this phase. Intra-operatively, the surgical team could implement evidence-based strategies to mitigate stress, such as intraoperative music therapy, virtual reality gaming for patients undergoing procedures under regional anesthesia, the administration of certain intraoperative NMDA receptor antagonists as anesthetic adjuvants, and the application of local anesthetic-impregnated dressings for wound care. Following surgical intervention, patients must be closely monitored within a structured post-operative framework to facilitate the early identification of psychiatric symptoms and ensure the timely delivery of indicated therapeutic support.

Strengths and Limitations

This scoping review possesses several methodological strengths. It comprehensively maps evidence for multiple post-operative psychiatric conditions across diverse surgical specialties, successfully capturing overarching clinical trends and localized variations. By incorporating a wide range of study designs, the review effectively details the breadth and depth of the existing literature while clearly delineating critical gaps in evidence. Furthermore, evaluating multiple concurrent mental health conditions provides a more holistic overview of post-surgical psychopathology, acknowledging that psychiatric morbidities frequently co-exist. The inclusion of studies published from 2000 to 2026 offers a comprehensive trajectory of how this research landscape has evolved over the last quarter-century, establishing a robust foundation for future investigations. Finally, adopting a highly systematic approach by adhering to the PRISMA-ScR reporting checklist ensures strict methodological transparency and reproducibility.

Nevertheless, this scoping review also has some limitations. The marked heterogeneity in study designs, assessment tools, and the chronological timing of evaluations precluded a direct comparison across investigations and prevented a formal meta-analytical synthesis of the findings. Furthermore, the exclusion of systematic reviews and studies focusing primarily on HRQOL may have constrained the overall scope of the mapped evidence. The restriction of our search to English-language publications may lead to the omission of relevant data published in other languages. Additionally, the inability to retrieve full-text articles for specific abstracts or to access papers published behind commercial paywalls is a potential source of selection bias that could impact the comprehensiveness of our findings. Finally, in strict accordance with the scoping review methodology, no formal critical appraisal or risk-of-bias assessment was performed on the included primary studies, which limits the ability to definitively gauge the methodological quality and underlying bias of the compiled evidence.

## Conclusions

Post-operative psychological disorders are prevalent among surgical patients and can significantly impair recovery trajectories and overall clinical outcomes. A multifaceted interaction exists between surgical interventions and mental health, mediated by psychosocial stressors, somatic symptoms, and individual patient vulnerabilities. Post-surgical psychological outcome optimization depends on a comprehensive care continuum, including rigorous individual risk assessment and mitigation, targeted pre-operative counseling, and evidence-based intraoperative strategies, such as the selection of optimal anesthetic and surgical techniques, use of neuroprotective adjuvants, and implementation of stress-reduction measures during regional anesthesia. Furthermore, ensuring meticulous post-operative monitoring to prevent surgical complications, maintaining a high index of clinical suspicion for psychiatric distress, and delivering timely psychosocial interventions are critical components of patient-centered care. Ultimately, the integration of structured mental health services into routine surgical care is essential to facilitate holistic patient recovery and enhance overall surgical success. Finally, there is an urgent need to develop and standardize specific mental assessment tools and reporting protocols for post-surgical patients.

## Appendices

Screening tools

Various screening tools were utilized to evaluate depression: Patient Health Questionnaire-9 (PHQ-9) [112], Hospital Anxiety and Depression Scale (HADS) [113], Beck Depression Inventory (BDI) [114], Primary Care Evaluation of Mental Health Disorders (PRIME-MD) [115], Self-Rating Depression Scale (SDS) [116], Hamilton Depression Rating Scale (HAMD) [117], Edinburgh Postnatal Depression Scale (EPDS) for postpartum depression [118], ICD-9-CM algorithms [119], Mini-International Neuropsychiatric Interview (MINI) [120], Brief Symptom Inventory (BSI) [121], Montgomery-Asberg Depression Rating Scale (MADRS) [122], Epidemiological Studies-Depression scale (CES-D) [123], Profile of Mood States (POMS) [124], Patient Global Impression of Change (PGIC) [125], and the Geriatric Depression Scale (GDS) [126].

Scales implemented for the assessment of anxiety: Hospital Anxiety and Depression Scale (HADS) [113], State-Trait Anxiety Inventory (STAI) or Spielberger [127], Hamilton Anxiety Rating Scale (HAMA) [128], Generalized Anxiety Disorder-7 (GAD-7) [129], Self-Rating Anxiety Scale (SAS) [130], Beck Anxiety Inventory (BAI) [131], Patient Global Impression of Change (PGIC) [125], Primary Care Evaluation of Mental Health Disorders (PRIME-MD) [115], Mini-International Neuropsychiatric Interview (MINI) [120], Profile of Mood States (POMS) [124], and the Brief Symptom Inventory (BSI) [121].

Instruments used to quantify PTSD symptoms: Post-Traumatic Stress Disorder Checklist for the Diagnostic and Statistical Manual of Mental Disorders, Fifth Edition (PCL-5) [132], the Mini-International Neuropsychiatric Interview (MINI) [120], and the Brief Symptom Inventory (BSI) [121].
